# Environmental and Social Factors Associated with the Occurrence of Severe Tungiasis and Scabies in the State of Ceará, Brazil: An Ecological Study

**DOI:** 10.3390/tropicalmed10050135

**Published:** 2025-05-16

**Authors:** Nathiel Silva, Carlos Henrique Alencar, Jorg Heukelbach

**Affiliations:** Postgraduate Course in Public Health, School of Medicine, Federal University of Ceará, Rua Prof. Costa Mendes 1608-5, Andar, Fortaleza 60430-140, Ceará, Brazil

**Keywords:** tungiasis, scabies, skin NTDs, control, One Health

## Abstract

Scabies and tungiasis are skin-related neglected tropical diseases (NTDs) associated with poverty and poor living conditions. We performed an ecological study covering a state in northeast Brazil to identify socio-economic and environmental factors associated with the occurrence of severe scabies and severe tungiasis, respectively. Data on disease occurrence on the municipality level were derived from a previous study based on online questionnaires. A total of 47 (26.0%) of the 181 state’s municipalities reported severe tungiasis, and 113 (62.4%) severe scabies. Municipalities with occurrence of severe tungiasis were characterized by higher annual rainfalls (median = 883 mm vs. 741 mm; *p* = 0.037), higher minimum temperatures (median = 23.4 °C vs. 22.7 °C; *p* = 0.002), higher aridity indices indicating more humid climates (median = 45.1 vs. 50.6; *p* = 0.019), lower altitudes (median = 88.8 m vs. 201 m; *p* < 0.001), higher mean air humidity (66.5% vs. 63%; *p* = 0.018), and better socioeconomic indices (Municipal Human Development Index [MHDI]—median = 0.616 vs. 0.611; *p* = 0.048/MHDI Longevity—mean = 0.769 vs. 0.759; *p* = 0.007/Municipal Development Index [MDI]—median = 27.5 vs. 21.8; *p* < 0.001). Municipalities with predominant luvisol soil characteristics had a lower risk for severe tungiasis (RR = 0.46; 95% CI = 0.27–0.79; *p* = 0.003), whereas municipalities with predominant gleysols had a significantly higher risk (RR = 2.44; 95% CI = 1.43–4.15; *p* = 0.010). Municipalities with occurrence of severe scabies were characterized by significantly higher annual rainfalls (median = 804 mm vs. 708 mm; *p* = 0.001), higher minimum temperatures (23.1 °C vs. 22.3 °C; *p* < 0.001), higher aridity index (median = 48.2 vs. 41.9; *p* = 0.014), higher air humidity (65.9% vs. 61%; *p* = 0.001), lower altitudes (median = 153 m vs. 246 m; *p* = 0.003), and better socio-economic indicators (MHDI—median = 0.616 vs. 608; *p*= 0.012/MHDI Education—mean = 0.559 vs. 0.541; *p* = 0.014/MDI—median = 24.3 vs. 21.1; *p* = 0.005). In multivariate regression analysis, MDI remained significantly associated with the presence of severe tungiasis in the final model (RR = 1.04; 95% CI: 1.02–1.05; *p* < 0.001) and the presence of severe scabies with minimum temperature (RR = 1.13; 95% CI: 1.04–1.24; *p* = 0.003) and aridity index (RR = 1.01; 95% CI: 1.00–1.01; *p* = 0.004). Our study underscores the importance of environmental and socioeconomic factors for the occurrence of severe scabies and tungiasis in a semi-arid climatic context, offering a perspective for identification of high-risk areas, and providing evidence for the control of skin NTDs withina One Health approach.

## 1. Introduction

Tungiasis is caused by penetration of the female sand flea *Tunga penetrans* into the skin of its host, leading to itching, pain, and, in the long-term, deformity of the toes and other complications. The disease is endemic in resource-poor urban and rural communities in endemic areas in sub-Saharan Africa, South America, and the Caribbean [1,2]. Domestic animals—such as dogs, cats, and pigs—but also rodents and sylvatic animals play an important role in transmission, and in the maintenance of the flea’s life cycle [3]. Tungiasis poses a substantial burden in endemic regions, where it may cause debilitation, chronic morbidity, and progress to deformation of digits and mutilation [1,4,5,6].

On the other hand, scabies is a contagious disease caused by the parasitic mite *Sarcoptes scabiei* var. *hominis*, causing itching and skin eruptions [7]. The disease shows particularly high prevalences in tropical and subtropical regions, mainly in resource-poor urban and rural communities. The transmission of scabies mites in these settings occurs via direct contact or fomites, and is embedded in a complex web of causation, including socio-economic determinants such as poor living conditions and low levels of education [7,8]. Risk factors for scabies include illiteracy, limited access to health services, overcrowding, migration and poor housing conditions, poor sanitation, and sharing of clothing and bedding [9,10,11,12].

In endemic areas, scabies is often associated with low disease awareness in the affected populations, and limited access to the health systems and, subsequently, effective treatment. It may result in intense itching and pain, leading to sleep disturbances, and impact the psychological well-being of affected individuals [9,12]. Secondary bacterial infections often cause impetigo, abscess formation, and lymphadenopathy. Group A streptococcal pyoderma as a consequence of severe scabies may lead to poststreptococcal glomerulonephritis and acute rheumatic fever [9,13].

Both scabies and tungiasis form part of the group of skin-related neglected tropical diseases (NTDs). Limited access to health services is a driving factor for high prevalences and ongoing transmission of both skin NTDs in many at-risk populations. The severity of disease and clinical characteristics in the described settings are usually different from those encountered in high-income settings, with higher prevalences and consequently more cases showing severe morbidity [1,2,7,9]. In addition, environmental factors play an important role in disease transmission, especially in the case of tungiasis, as the parasite’s life cycle includes off-host stages (eggs are shed by embedded fleas which develop into larvae in the environment) [4,14].

Both skin NTDs have been included in the World Health Organization’s (WHO) updated roadmap for controlling NTDs and are increasingly being perceived as important public health issues in endemic communities. The WHO roadmap confirmed the need for more scientific data as a basis for evidence-based control measures [15]. In fact, systematic epidemiological data on the incidence and prevalence of tungiasis and scabies, and their associated environmental and social factors, are scarce. With the objective to provide data for elaboration of evidence-based disease control, measures, we performed an ecological analysis in a state in northeast Brazil, assessing socio-economic and environmental factors associated with severe tungiasis and severe scabies.

## 2. Materials and Methods

### 2.1. Study Area and Design

**Figure 1:**
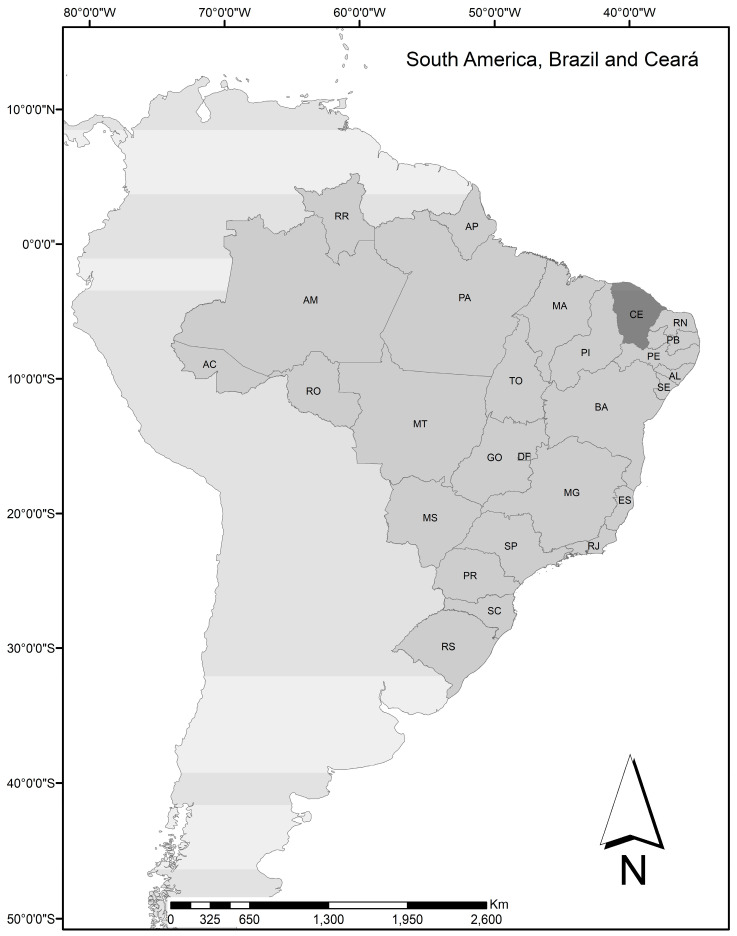
Location of Ceará State in northeast Brazil.

Ceará State is situated in northeast Brazil and has around 9.2 million inhabitants (Figure 1). There are 184 municipalities in the state, with slightly differing socio-economic, geographic, and climatic characteristics: an Atlantic coastal strip, predominantly marked by semi-arid climates with limited and irregular rainfalls; municipalities in the savannah region; and others in mountainous regions, partly covered by Atlantic rainforest.

We performed an ecological study with municipalities as the units of analysis, including all municipalities of the state. We aimed to identify sociodemographic, geographic, and environmental characteristics associated with the occurrence of severe cases of scabies and tungiasis in the municipalities.

### 2.2. Variables and Data Sources

Data on the occurrence of both diseases on the municipality level were obtained from a previous assessment, which was based on online questionnaires, to be completed by health professionals (data collection March–September 2021). Data collection and processing have been described in detail elsewhere [16,17,18]. In brief, information on the occurrence of tungiasis and scabies cases in the municipalities was based on the answers and comments provided by health professionals via an online platform. The occurrence of severe cases was asked in a separate question and thus based on the direct responses provided by professionals, considering their routines and clinical practices. The online questionnaires presented images of severe cases as a reference for the respondents. In addition, municipalities with the occurrence of severe cases were identified based on details provided by respondents in a dedicated questionnaire field describing observed disease manifestations (i.a., hospital interventions, involvement of multiple areas of the body, bacterial superinfections, and the development of disabilities).

In three of the state’s municipalities, there were no responses from health professionals for each disease, respectively, and consequently, no information on scabies and tungiasis occurrence was available from these municipalities (namely Missão Velha, Moraújo and Paramoti for scabies, and Iracema, Lavras da Mangabeira and Várzea Alegre for tungiasis). Thus, 181 (98.3%) municipalities were included in data analyses for scabies and tungiasis, respectively.

Environmental and socio-economic explanatory variables, and their data sources, are presented in Table 1. All data were retrieved from official information from the government and reference research institutions.

We analyzed the association of these explanatory variables with two different outcomes: occurrence of severe tungiasis and occurrence of severe scabies. As tungiasis and scabies were reported in the majority of municipalities (and in fact can be assumed to occur in virtually all municipalities), and as interventions should focus primarily on areas and factors associated with severe forms of the diseases, for this study we defined occurrence of severe cases of the respective disease in the municipality as the outcome.

### 2.3. Statistical Analyses

Bivariate analysis was performed using the *t*-test and Mann–Whitney test for continuous variables, as well the Chi-squared and Fisher tests for categorical variables, where applicable. Data normality was tested using the Shapiro–Wilk test. The Welch test was used as an alternative to the *t*-test when there was a violation of the assumption of homogeneity of variance by Levene test. We used the statistical software Jamovi 2.5 (Jamovi Project 2024).

Predominant soil characteristics were only included for severe tungiasis, considering development of off-host stages in the soil during the flea’s life cycle. Official state data indicate the types of soil that predominate in each municipality. Thus, each observation unit generated data on the presence or absence of each soil, which was then analyzed in our model.

In addition, a Poisson backward regression model with robust variance was used to adjust for potential confounding effects and to identify possible independent interactions between explanatory variables and the outcomes. The goodness of fit test was performed to identify the presence of overdispersion in the final model. Variables with a *p*-value < 0.05 were considered significantly associated. In the final model, only statistically significant variables remained. Risk ratios and their respective 95% confidence intervals were calculated. Prior to multivariable modeling, collinearity among independent variables was assessed. Variance inflation factors (VIF), tolerance values, partial R-squared, and condition indices were examined. Collinearity was considered high if VIF values exceeded 10, tolerance values were below 0.1, or in the case of condition indices > 30. Variables with excessive collinearity were reviewed based on theoretical relevance.

## 3. Results

A total of 47/181 (26.0%) municipalities reported occurrence of severe tungiasis, and severe scabies was reported from a total of 113/181 (62.4%) municipalities.

**Figure 2:**
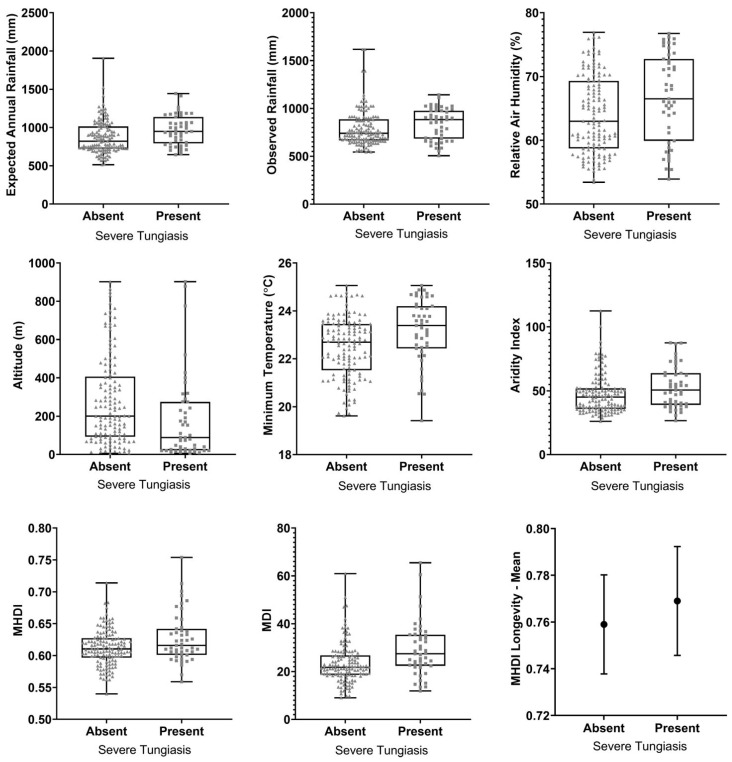
Dispersion of data for variables significantly associated with tungiasis. Box plots indicate medians, interquartile ranges, and amplitudes, with each dot indicating a municipality. Bar charts indicate means and standard deviations.

Table 2 presents socioeconomic and environmental variables associated with severe tungiasis. Within the mainly semiarid context of Ceará State, municipalities with occurrence of severe tungiasis were characterized by significantly higher rainfalls, higher temperatures, higher aridity indices (i.e., lower aridity), lower altitudes, and better socio-economic indices. The dispersion of data for variables significantly associated with severe tungiasis is presented in Figure 2.

Municipalities with predominant luvisol soil characteristics had a significantly lower risk for severe tungiasis (RR = 0.46; 95% CI = 0.27–0.79; *p* = 0.003), whereas municipalities with predominant gleysol characteristics had a significantly higher risk (RR = 2.44; 95% CI = 1.43–4.15; *p* = 0.010).

**Figure 3:**
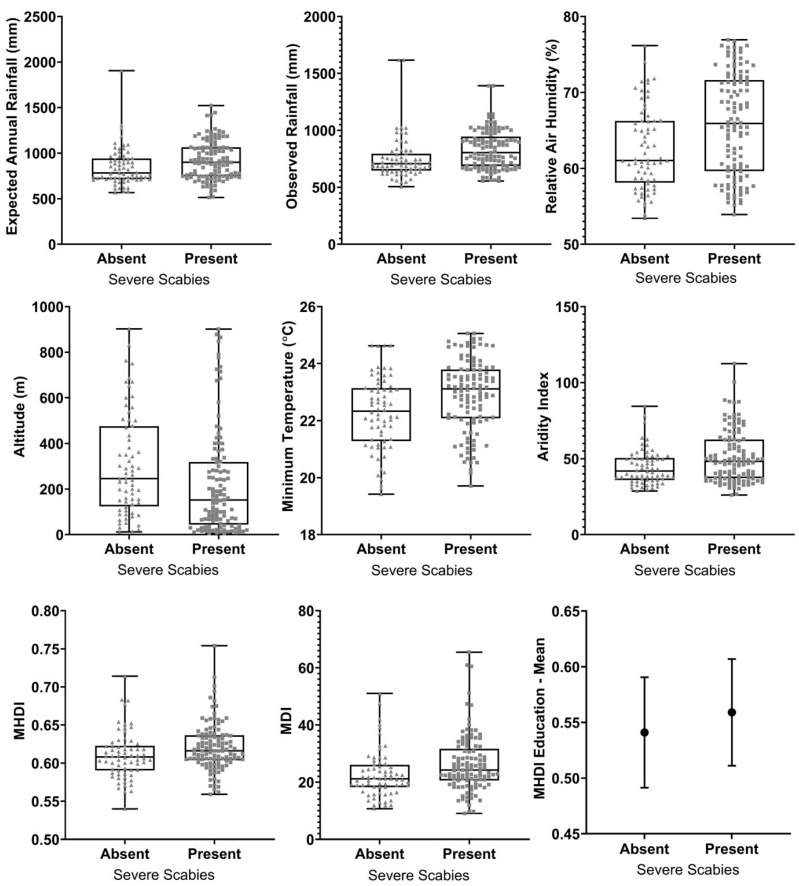
Dispersion of data for variables with statistically significant associations for severe scabies in the study. Box plots indicate medians, interquartile ranges, and amplitudes, with each dot indicating a municipality. Bar charts indicate means and standard deviations.

Socioeconomic and environmental variables associated with severe scabies are presented in Table 3. Municipalities with occurrence of severe scabies were characterized by significantly higher rainfalls and higher temperatures, but with higher altitudes and higher aridity indices (indicating lower aridity and temperature). The dispersion of data for variables significantly associated with severe scabies is presented in Figure 3.

After assessing collinearity between exposure variables, multicollinearity was identified, though not necessarily between simple pairs of variables. As a result, collinear variables with high VIF were excluded from the initial model. Both models did not present overdispersion (tungiasis model—*p* = 0.999; scabies model—*p* = 1.000). Among the climate-related indicators, air humidity, observed rainfall, and expected rainfall were excluded from multivariate analyses, due to their strong correlation with the aridity index. Among the socioeconomic variables, the municipal Human Development Index (MHDI) was excluded due to its collinearity with the Global Municipal Development Index (MDI). In the case of tungiasis, MDI remained significantly associated with the presence of severe disease (RR = 1.03; 95% CI: 1.00–1.05; *p* = 0.038); altitude (RR = 0.99; 95% CI: 0.997–1.00; *p* = 0.69) and minimum temperature (RR = 1.14; 95% CI: 0.72–1.82; *p* = 0.57) remained in the pre-final model. After the exclusion of non-significant variables, only MDI was kept in the final model (RR = 1.04; 95% CI: 1.02–1.05; *p* < 0.001).

In the case of scabies, three variables remained in the pre-final model: minimum temperature (RR = 1.13; 95% CI: 1.04–1.24; *p* = 0.006); aridity index (RR = 1.01; 95% CI: 1.00–1.02; *p* = 0.04); and MDI (RR= 1.002; 95% CI: 0.99–1.01; *p* = 0.753). The final model including only statistically significant variables consisted of the aridity index (RR = 1.01; 95% CI: 1.00–1.01; *p* = 0.004) and minimum temperature (RR = 1.13; 95% CI: 1.04–1.24; *p* = 0.003).

## 4. Discussion

Our study shows that both severe tungiasis and severe scabies were associated with environmental and socio-cultural determinants in an endemic area of major geographic extension. The study covered an entire state, and, to the best of our knowledge, is the first study of this kind assessing scabies and tungiasis. Within the semi-arid context of Ceará State, municipalities with higher rainfalls, higher temperatures, more humid climates, and lower altitudes more frequently reported the occurrence of severe tungiasis. Similarly, the occurrence of severe scabies in the municipalities was somehow linked to higher temperatures, lower altitudes, and more humid climates.

Municipalities with higher social development indices, and consequently with better diagnostic and treatment facilities and better health systems, more commonly reported severe tungiasis and scabies. In Ceará State, municipalities with better socio-economic conditions are usually bigger cities, and harbor a considerable number of vulnerable populations living in subnormal urban agglomerations (such as so-called favelas). This favors the occurrence of the diseases that are strongly linked to populations living in poverty, under conditions of precarious housing and overcrowding.

In general, a lower gross domestic product (GDP) per capita correlates with higher Disability-Adjusted Life Years (DALYs) for dermatology-related infectious diseases, driven by limited resources and access to the health system [19]. Some studies across different countries indicate that scabies occurrence is primarily driven by geographic and behavioral factors, and that the number of cases is rising in high-income regions, influenced by lifestyle and overcrowding: for example, in nursing homes [20,21]. However, this situation is not comparable to our study area and other resource-poor settings in tropical regions, where scabies transmission dynamics differ from wealthy areas, and occurrence and severity of disease are linked to community transmission [8,12,22]. Our findings that severe scabies was observed more commonly in municipalities with higher socio-economic indices indicates that these municipalities are usually bigger cities in Ceará and thus there were more responses from health care workers, increasing the odds of positive responses, in addition to the occurrence of a higher amount of sub-normal housing and crowded conditions on the outskirts.

Tungiasis is intrinsically associated with poverty, disproportionately affecting rural and urban communities. On the other hand, tungiasis is a focal disease, and prevalences vary widely, even among neighboring areas in the same municipality, with similar risk profiles, reflecting the complex interplay of socio-economic factors, animal reservoirs, and individual behavior. Evidence indicates that tungiasis does not only mirror poverty but may perpetuate it, driven by inadequate housing, poor hygiene, low socioeconomic status, and limited health access to health care, leading also to school absenteeism [1,6,10,23].

The climate significantly affects infectious diseases like scabies and tungiasis, with rainfall and temperature shifts influencing the parasite’s life cycle and survival. *Sarcoptes scabiei* mites survive longer in cooler, humid conditions but dehydrate faster at higher temperatures. We have recently described how 93% of municipalities in Ceará State reported seasonal variations in scabies cases, spanning diverse climates from hot and dry to humid and coastal [18]. While in some regions, scabies outbreaks peak during the cooler months (e.g., due to increased crowding of people during the rainy season, more common use of clothing, limited ventilation of the skin, and impossibility of drying clothing and bedding), tropical and subtropical areas show no clear seasonal trends, suggesting a need for further investigation [1,24]. However, climatic differences between seasons and regions are relatively low in our study area, and not comparable to regions with moderate climates. In Taiwan, where subtropical, tropical, and moderate climates prevail, scabies incidence was inversely correlated with temperature and positively correlated with humidity, which suggests that cool and humid conditions can increase the survival and reproductive success of mites, contributing to the dynamics of scabies transmission [25].

The development of off-host stages of *T. penetrans* is influenced by environmental factors such as humidity, temperature, and soil composition, as well as human-related factors such as livestock density and pet ownership [4,14,26,27]. However, these determinants alone do not fully explain the geographical distribution of tungiasis, as there are few studies on this topic, highlighting the need for more comprehensive research to better understand spatial risk patterns.

Previous studies have shown that tungiasis prevalence increased considerably during dry seasons, thriving in areas with temperatures of 18.3 °C to 26.4 °C, peaking at 26.9 °C, and 73.1% humidity, as well aridity index average of 55.19, which is in line with our findings [4,27]. Ecological niche modeling in Latin America identified forests, croplands, and shrublands at an average elevation of 380 m were found to be suitable for tungiasis [4].

Tungiasis transmission dynamics are closely tied to development of pre-adult off-host stages in the soil. In Ceará, Brazil, rural communities use soil irrigation to reduce infestations, highlighting seasonal community-based prevention strategies [28]. In our study area, the predominant soil characteristics were neosols (36.00%), argisols (24.7%), and luvisols (16.7%), with sandy, clay, and laterite particularities. Severe tungiasis occurred less commonly in municipalities with predominant luvisols, whereas the opposite was the case in municipalities characterized by predominant gleysols. These findings emphasize the need for further research on soil exposure and its role in disease patterns across regions and seasons. High temperatures and direct sunshine reduce the survival of eggs and larvae in the soil, reducing the positivity rates in outdoor samples; indoor environments, on the other side, showed a 3.7-fold higher likelihood of positive soil samples, highlighting their critical role in transmission [14,29,30].

Epidemiologists, public health professionals, and policy makers should also anticipate shifts in disease patterns driven by climate instability, often exacerbated by poverty and overcrowding. The production and availability of relevant educational materials for teachers and health professionals is essential, with schools and communities playing a pivotal role in disease control, especially in impoverished areas. As pilot projects expand, strategies should be refined and shared to support WHO guidelines and integrate control into national health plans [2,7,15,16,31]. Mapping, implementation, and surveillance are critical, while vaccine remains a promising ongoing research area for scabies [32].

Our study is subject to limitations, including the potential for ecological bias due to variations within municipalities in size, population density, geomorphology, climate, urbanization levels, and social indicators. This heterogeneity and high degree of collinearity of several variables may lead to misleading associations, especially between socio-environmental factors and the diseases studied, limiting the applicability of the multiple regression model, and consequently, some variables with significant associations in the bivariate analyses lost statistical significance in multivariate analyses. Considering this, in fact, the variables which remained in the final model are somehow interchangeable with their collinear variables, and thus interpretation of multivariate analysis is limited in our specific case. The capital city Fortaleza has affluent neighborhoods alongside impoverished favelas, while another major city in the metropolitan region of Fortaleza (Caucaia) features dense urban centers, coastal areas, mountains, and drylands. These varied contexts demand caution in interpreting the results. In addition, the simple fact that a higher number of people are present in a certain municipality may have increased the odds for positive responses regarding incidences of severe tungiasis and severe scabies. Consequently, the completeness and correctness of the data provided by health professionals may differ, and data may have been more accurate coming from bigger and more developed municipalities. Based on our study design, severe disease could not be defined based on clear-cut clinical criteria but was mainly based on the personal experience of the respondents and on sample photographs, which may have led to the underreporting of severe disease.

Considering the ecological nature of our study, several sources of confounding and high collinearity of variables, the results should be considered with care and be confirmed by future studies. Further studies should be performed, using individualized data, to substantiate and confirm our study results on factors associated with the occurrence of severe scabies and tungiasis, respectively.

## 5. Conclusions

Our study highlights the significant role of environmental determinants in the occurrence of severe scabies and severe tungiasis. Interdisciplinary measures—bringing together policy makers, community leaders, schoolteachers, physicians, veterinarians, and other social actors—are essential to elaborate effective and sustainable control strategies [33]. While environmental management is an important aspect of disease control, it must be part of this comprehensive One Health control approach that also includes individual treatment of humans, reduction in animal reservoirs, public health interventions, and campaigns to increase the visibility of skin NTDs. Strengthening leadership, securing funding, and developing low-cost, rapid assessment tools, along with the use of new technologies for accurate field diagnosis of both scabies and tungiasis, are essential for effective disease management [2,9,12].

Our study presents insights and systematized data on two skin NTDs across a large geographical area, enabling the identification of high-risk areas. This low-cost approach could also be replicated for other diseases, regions, and countries, further supporting national and international collaborations. With intensifying global climate changes, there is an urgent need to study transmission dynamics in more detail, especially in those regions that are most affected by climate change.

## Figures and Tables

**Table 1:** Explanatory variables and data sources.

Variable	Data Source	Source/Website	Comments
**Environmental Variables**			
Expected annual rainfall (mm)	FUNCEME	https://chuvas.funceme.br/mensal/municipios/media/2025	rainfall forecast for 2021 in the municipality
Rainfall observed (mm)	FUNCEME	https://chuvas.funceme.br/mensal/municipios/media/2025	amount of rainfall observed during 2021 in the municipality
Average temperature (°C)	Climate-data	https://en.climate-data.org/south-america/brazil/ceara-212/	mean of the mean temperatures in the municipality between 1991 and 2021
Minimum temperature (°C)	Climate-data	https://en.climate-data.org/south-america/brazil/ceara-212/	mean of the minimum temperatures in the municipality between 1991 and 2021
Maximum temperature (°C)	Climate-data	https://en.climate-data.org/south-america/brazil/ceara-212/	mean of the maximum temperatures in the municipality between 1991 and 2021
Aridity Index	IPECE	https://www.ipece.ce.gov.br/indice-municipal-de-alerta/	ratio between the mean annual rainfall and mean annual evapotranspiration in 2021 (humid: ≥ 1; most subhumid: 0.65 ≤ AI < 1; dry sub-humid 0.50 ≤ AI < 0.65; semiarid 0.20 ≤ AI < 0.50; hyperarid < 0.20
Altitude	IPECE	http://ipecedata.ipece.ce.gov.br/ipece-data-web/	altitude of the downtown area/city center
Predominant soil	IPECE	http://ipecedata.ipece.ce.gov.br/ipece-data-web/	most common soil type in the municipality (argisols, neosols, luvisols, latosols, planosols, plinthosoil, vertisols, nitisols, chernosols, cambisols, gleysols, organosols)
Average relative humidity	Climate-data	https://en.climate-data.org/south-america/brazil/ceara-212	mean of relative air humidity in the municipality between 1991 and 2021
**Socioeconomic Variables**			
Municipal Human Development Index (MHDI) in 2010-Global	IPECE	http://ipecedata.ipece.ce.gov.br/ipece-data-web/	living conditions of a municipality’s population—composed of 3 indicators (GNI per capita, years of schooling, life expectancy at birth)
- MHDI Income	IPECE	http://ipecedata.ipece.ce.gov.br/ipece-data-web/	Municipal Human Development Index considering GNI per capita
- MHDI Education	IPECE	http://ipecedata.ipece.ce.gov.br/ipece-data-web/	Municipal Human Development Index considering only years of schooling
- MHDI Longevity	IPECE	http://ipecedata.ipece.ce.gov.br/ipece-data-web/	Municipal Human Development Index considering only life expectancy at birth
Municipal Development Index (MDI) in 2018	IPECE	http://ipecedata.ipece.ce.gov.br/ipece-data-web/	levels of development achieved by municipalities (thirty indicators classified into four groups). This index can be used to monitor the development conditions of municipalities, acting as a diagnostic tool and a reference for proposing and guiding public policies.
Social Vulnerability Index (SVI) in 2010	IPEA	https://ivs.ipea.gov.br/#/repositorio	lack or insufficiency of assets capable of enabling well-being or social bonds that ensure social protection (i.a. income, housing, drink water supply, basic sanitation, access to health services, access to education).
Social Progress Index (SPI) in 2024	SPI Brazil	https://ivs.ipea.gov.br/#/repositorio	multidimensional overview of the performance of municipalities in meeting the social and environmental needs of their citizens (54 indicators of three dimensions: basic human needs, foundations of well-being, and opportunity to progress)

FUNCEME (Ceará Meteorology and Water Resources Foundation); IPECE (Instituto de Pesquisa e Estratégia Econômica do Ceará); IPEA (Institute for Applied Economic Research); SPI Brazil (IPS Brasil, a group formed by the collaboration between the Amazon Institute for Man and the Environment (Imazon), the Avina Foundation, the Amazon Entrepreneurship Center, the Amazon 2030 Initiative, Anattá—Research and Development, and the Social Progress Imperative).

**Table 2:** Bivariate analysis of variables associated with severe tungiasis in Ceará State.

Variables	Mean	Standard Deviation	Quartile 25%	Median	Quartile 75%	*p* Value
Enviromental Variables						
** Expected annual rainfall (mm)						
*Severe tungiasis absent*	-	-	724	821	1009	0.002
*Severe tungiasis present*	-	-	798	950	1101	
** Rainfall observed (mm)						
*Severe tungiasis absent*	-	-	668	741	880	0.037
*Severe tungiasis present*	-	-	684	883	976	
** Mean temperature (°C)						
*Severe tungiasis absent*	-	-	25.9	26.6	27.2	0.217
*Severe tungiasis present*	-	-	26.5	26.8	27.2	
** Minimum temperature (°C)						
*Severe tungiasis absent*	-	-	21.5	22.7	23.4	0.002
*Severe tungiasis present*	-	-	22.4	23.4	24.2	
** Maximum temperature (°C)						
*Severe tungiasis absent*	-	-	30.5	31.6	32.3	0.475
*Severe tungiasis present*	-	-	30.0	31.4	32.4	
** Aridity Index						
*Severe tungiasis absent*	-	-	36.0	45.1	51.8	0.019
*Severe tungiasis present*	-	-	39.4	50.6	63.1	
** Altitude (downtown)						
*Severe tungiasis absent*	-	-	94.3	201	402	<0.001
*Severe tungiasis present*	-	-	24.0	88.8	259	
** Mean of relative air humidity						
*Severe tungiasis absent*	-	-	58.8	63.0	69.1	0.018
*Severe tungiasis present*	-	-	60.5	66.5	72.6	
Socioeconomic Variables						
** Municipal Human Development Index Global						
*Severe tungiasis absent*	-	-	0.597	0.611	0.627	0.048
*Severe tungiasis present*	-	-	0.603	0.616	0.641	
* Municipal Human Development Index Education						
*Severe tungiasis absent*	0.548	0.0473	-	-	-	0.058
*Severe tungiasis present*	0.563	0.0518	-	-	-	
* Municipal Human Development Index Longevity						
*Severe tungiasis absent*	0.759	0.0212	-	-	-	0.007
*Severe tungiasis present*	0.769	0.0233	-	-	-	
** Municipal Human Development Index Income						
*Severe tungiasis absent*	-	-	0.530	0.551	0.574	0.088
*Severe tungiasis present*	-	-	0.536	0.564	0.589	
** Municipal Development Index						
*Severe tungiasis absent*	-	-	18.7	21.8	26.6	<0.001
*Severe tungiasis present*	-	-	22.6	27.5	35.2	
*** Social Vulnerability Index						
*Severe tungiasis absent*	0.439	0.0534	-	-	-	0.913
*Severe tungiasis present*	0.440	0.0706	-	-	-	
* Social Progress Index						
*Severe tungiasis absent*	56.6	2.63	-	-	-	0.215
*Severe tungiasis present*	57.2	3.28	-	-	-	

* *t*-Test; ** Mann–Whitney test; *** Welch test.

**Table 3:** Bivariate analysis of variables associated with severe scabies in Ceará State.

Variables	Mean	Standard Deviation	Quartile 25%	Median	Quartile 75%	*p* Value
Enviromental Variables						
** Expected annual rainfall (mm)						
*Severe scabies absent*	-	-	717	782	938	0.006
*Severe scabies present*	-	-	747	900	1064	
** Rainfall observed (mm)						
*Severe scabies absent*	-	-	648	708	791	<0.001
*Severe scabies present*	-	-	689	804	930	
** Mean temperature (°C)						
*Severe scabies absent*	-	-	25.6	26.6	27.1	0.174
*Severe scabies present*	-	-	26.3	26.8	27.2	
** Minimum temperature (°C)						
*Severe scabies absent*	-	-	21.3	22.3	23.1	<0.001
*Severe scabies present*	-	-	22.1	23.1	23.8	
** Maximum temperature (°C)						
*Severe scabies absent*	-	-	30.5	31.6	32.3	0.642
*Severe scabies present*	-	-	30.3	31.6	32.4	
** Aridity Index						
*Severe scabies absent*	-	-	36.0	41.9	50.3	0.014
*Severe scabies present*	-	-	37.3	48.2	62.5	
** Altitude (downtown)						
*Severe scabies absent*	-	-	130	246	472	0.003
*Severe scabies present*	-	-	48.0	153	317	
** Mean of relative air humidity						
*Severe scabies absent*	-	-	58.3	61.0	66.3	0.001
*Severe scabies present*	-	-	59.7	65.9	71.5	
Socioeconomic Variables						
** Municipal Human Development Index Global						
*Severe scabies absent*	-	-	0.591	0.608	0.622	0.012
*Severe scabies present*	-	-	0.604	0.616	0.636	
* Municipal Human Development Index Education						
*Severe scabies absent*	0.541	0.0496	-	-	-	0.014
*Severe scabies present*	0.559	0.0479	-	-	-	
* Municipal Human Development Index Longevity						
*Severe scabies absent*	-	-	0.746	0.761	0.776	0.958
*Severe scabies present*	-	-	0.749	0.760	0.775	
** Municipal Human Development Index Income						
*Severe scabies absent*	-	-	0.530	0.549	0.567	0.055
*Severe scabies present*	-	-	0.540	0.562	0.583	
** Municipal Development Index						
*Severe scabies absent*	-	-	18.3	21.1	25.7	0.005
*Severe scabies present*	-	-	20.6	24.3	30.9	
*** Social Vulnerability Index						
*Severe scabies absent*	0.440	0.0609	-	-	-	0.856
*Severe scabies present*	0.438	0.0568	-	-	-	
* Social Progress Index						
*Severe scabies absent*	56.66	2.8512	-	-	-	0.613
*Severe scabies present*	56.88	2.8071	-	-	-	

* *t*-test; ** Mann–Whitney test; *** Welch test.

## Data Availability

The raw data supporting the conclusions of this article will be made available by the authors on request.
